# Causal Effects of Genetically Predicted Cystatin C on Osteoporosis: A Two-Sample Mendelian Randomization Study

**DOI:** 10.3389/fgene.2022.849206

**Published:** 2022-05-02

**Authors:** Jiaqin Yuan, Lipeng Peng, Fujun Luan, Jie Li, Jinglin Zhang, Wei Jiang, Wenting Wang

**Affiliations:** ^1^ Department of Orthopedics, The Second People’s Hospital of Yibin, Yibin, China; ^2^ Department of Orthopedics, Yibin Hospital, West China Hospital of Sichuan University, Yibin, China; ^3^ Trauma Orthopaedics, Yongchuan Hospital of Chongqing Medical University, Chongqing, China; ^4^ Public Health, Chongqing Medical University, Chongqing, China; ^5^ Department of Anesthesiology, The Second Affiliated Hospital of Hainan Medical University, Haikou, China

**Keywords:** mendelian randomization, cystatin C, osteoporosis, genetics, bone mineral density

## Abstract

**Objectives:** Although it has long been reported that high levels of cystatin C could contribute to the development of osteoporosis in some studies, no evidence has established a causal association between them thus far.

**Methods:** A Mendelian randomization (MR) study was conducted to determine the causal effect of cystatin C on osteoporosis based on public databases obtained from separately published genome-wide association studies (GWASs). The single-nucleotide polymorphisms (SNPs) for cystatin C were extracted from the MR-Base (CKDGen, 33,152 participants), and the SNPs for osteoporosis were extracted from the United Kingdom Biobank project (United Kingdom Biobank, including 5,266 osteoporosis cases and 331,893 controls). We defined the odds ratio (OR) of IVW methods as the primary outcome. In addition, weighted median and MR–Egger regressions were used in the sensitivity analysis.

**Results:** In IVW, we found that genetically predicted cystatin C was causally associated with the risk of osteoporosis with an OR of 1.02 [95% confidence interval (CI) = 1.003–1.025, *p* = 0.01]. In the further sensitivity analysis, weighted median regression also showed directionally similar estimates (OR = 1.02, 95% CI = 1.005–1.03, *p* = 0.005), and MR–Egger regression (OR = 1.02, 95% CI = 1.000–1.036, *p* = 0.15) revealed similar estimates but with lower precision. The funnel plot, MR–Egger intercept, and MR-PRESSO all indicate that no directional pleiotropic effect was observed.

**Conclusion:** In conclusion, our MR study showed evidence of a causal association between serum cystatin C levels and osteoporosis, which also needs to be verified by studies with larger sample sizes in the future. Early monitoring of cystatin C may enable us to prevent osteoporosis-related diseases.

## 1 Introduction

Osteoporosis is a slowly progressing systemic metabolic bone disease that is directly caused by increased bone resorption and decreased bone formation. The typical features are mainly bone loss, bone microstructure destruction, increased bone fragility, and fragility fractures. Osteoporosis can greatly increase the risk of osteoporotic fractures, mainly in the forearm, hip, and lumbar spine (Golob and Laya, 2015). According to reports, the prevalence rate of osteoporosis in people over 50 years old in China was 19.2%, and the prevalence rate of osteoporosis among people over 65 years old reached 32.0% in 2018 (Gao et al., 2021). The condition greatly increases the economic costs of individuals and the whole country, which highlights the importance of taking measures to curtail the risk of osteoporosis.

The prevalence of osteoporosis increased significantly with increasing age, leading to a higher risk of fragility fractures. In addition, osteoporosis usually coexists with diseases such as hypertension and diabetes (Gutzwiller et al., 2018). Therefore, early diagnosis and prevention are extremely important. Studies have shown that chronic kidney disease (CKD) is an independent risk factor for osteoporosis (Najar et al., 2017). Patients with renal insufficiency have a significantly lower bone mineral density (BMD) (Tseng et al., 2014; Bezerra de Carvalho et al., 2019). Serum cystatin C is considered a sensitive indicator of early renal insufficiency. Because cystatin C is less dependent on muscle mass, it is better than the measurement of renal function based on serum creatinine. A large-scale cohort study by Tanaka et al. (Tanaka et al., 2019) showed that serum cystatin C is significantly increased in osteoporosis, and cystatin C levels of ≥ 0.840 mg/L indicate the presence of osteoporosis. However, some unmeasured risks may affect the causal inferences of observational studies, such as physical activity, lifestyle habits, or other underlying diseases. Therefore, observational studies, including cohort studies, can only explore the association between risk factors and diseases but not the causal effect.

**FIGURE 1:**
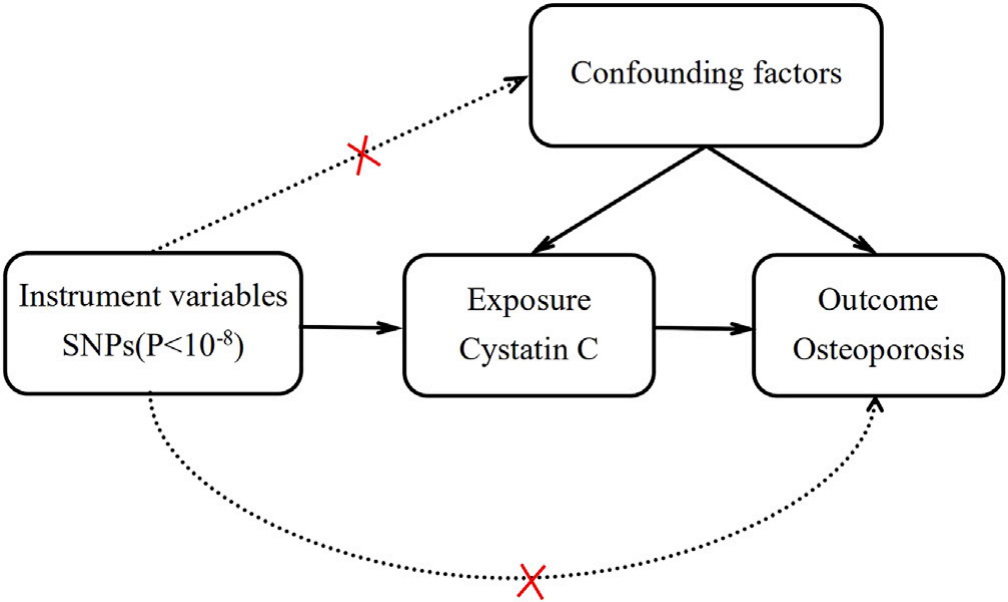
The illustrative diagram of two-sample Mendelian randomization analysis.

Mendelian randomization (MR) is an emerging research method for inferring potential causality that uses single-nucleotide polymorphisms (SNPs) as instrumental variables (IVs) to assess the causal association between exposure factors and outcomes (Emdin et al., 2017; Davies et al., 2018). Since SNP alleles are assorted randomly during gamete formation, all the inherited genetic variants have been determined at conception. Therefore, MR is not susceptible to potential confounding factors and measurement errors, and it can be used as a credible tool to infer causality (Figure 1).

This study aimed to assess the causal association between serum cystatin C levels and osteoporosis by using a two-sample MR method.

## 2 Materials and Methods

### 2.1 Overall Study Design

All of our data were obtained from published studies, which were supported by the institutional review committee, and informed consent was obtained from the participants in their original research (Lawlor, 2016; Richmond et al., 2016). Thus, further sanctions were not needed (Tan et al., 2021a). Two-sample MR was used to explore the causal association between serum cystatin C and the risk of osteoporosis with the definition of single-nucleotide polymorphisms (SNPs) as IVs.

### 2.2 Data Sources

#### 2.2.1 Genetic Instrument Variants for Exposure

Summary statistics on cystatin C were retrieved from the publicly available genome-wide association study (GWAS) summary data sources on the MR-Base platform, which included 33,152 individuals of European ancestry (Pattaro et al., 2016). The criteria we selected for the SNPs were as follows: 1) The SNPs were highly correlated with cystatin C with genome-wide significance (*p* < 5×10^−8^). 2) The SNPs were independent of each other to avoid offsets caused by linkage disequilibrium (LD), and the LD of SNPs associated with cystatin C must meet the *r* ^2^ < 0.001, window size =10,000 kb. LD levels were estimated from the 1000 Genomes Project (1000 Genomes Project Consortium et al., 2010) based on European samples. 3) To ensure a strong correlation between instrumental variables and exposure factors, the F statistic of SNPs was usually used to judge the strength of the correlation. When the F statistic >10, it is generally considered that there is no bias of weak instrumental variables. F statistics = (*β*/SE)^2^.

#### 2.2.2 Study Outcome: Osteoporosis

Data on osteoporosis were obtained from the United Kingdom Biobank (UKB), which is available at https://gwas.mrcieu.ac.uk/datasets/ukb-a-87/ and includes 5,266 osteoporosis cases and 331,893 controls of European ancestry.

### 2.3 Statistical Analysis

MR analysis was performed by R software (version 4.1.2, http://www.r-project.org) and the TwoSampleMR package (version 0.5.6) (Broadbent et al., 2020). The data and codes of this study from the corresponding author can be obtained from the corresponding author upon reasonable request.

Traditional inverse variance weighting (IVW), was used to assess the association between genetically predicted cystatin C levels and the risk of osteoporosis (Burgess et al., 2013). However, IVW is based on the premise that all instrumental variables are valid. As long as one SNP does not satisfy the assumption of instrumental variables, bias will be generated. Therefore, weighted median (Bowden et al., 2016) and MR–Egger (Bowden et al., 2015) were used for additional sensitivity analysis. The weighted median method requires at least 50% of the SNPs to meet the premise of valid instrumental variables. After the included SNPs are arranged according to weight, the median of the corresponding distribution function is obtained as the result of our analysis. MR–Egger regression can provide a valid effect estimate if genetic instruments do not rely on pleiotropic effects. The intercept estimated by MR–Egger was used to evaluate pleiotropic effects. If the intercept test of MR–Egger is not significantly different from zero, then there is no evidence for directional pleiotropic effects. In addition, we used MR Pleiotropy RESidual Sum and Outlier (MR-PRESSO) to remove SNPs with pleiotropic outliers (*p* < 0.1) (Verbanck et al., 2018). Of, if the following three conditions were met, we believed that there was a significant causal association between cystatin C levels and osteoporosis: 1) There was a significant difference in the IVW method (*p* < 0.05), 2) the estimation directions of the IVW, weighted median, and MR–Egger methods were consistent, and 3) neither the MR–Egger intercept test nor the MR-PRESSO global test was significant (*p* > 0.05) (Zhang et al., 2021).

## 3 Results

### 3.1 Instrumental Variables for Cystatin C

**FIGURE 2:**
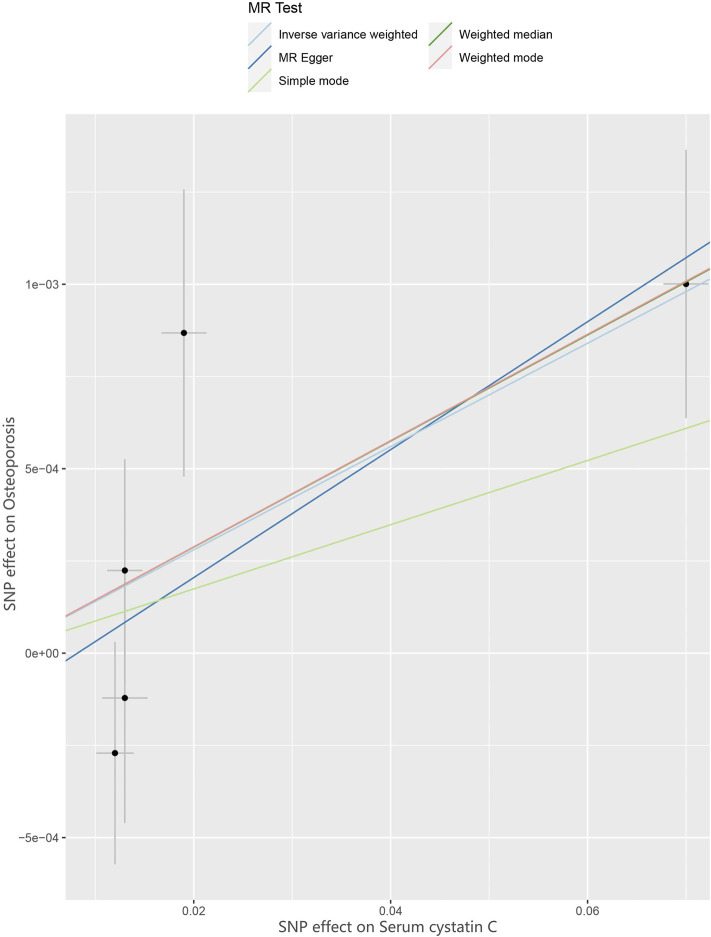
Scatter plot to visualize causal effect of Cystatin C on osteoporosis risk. The slope of the straight line indicates the magnitude of the causal association. IVW indicates inverse-variance weighted; and MR, Mendelian randomization.

**FIGURE 3:**
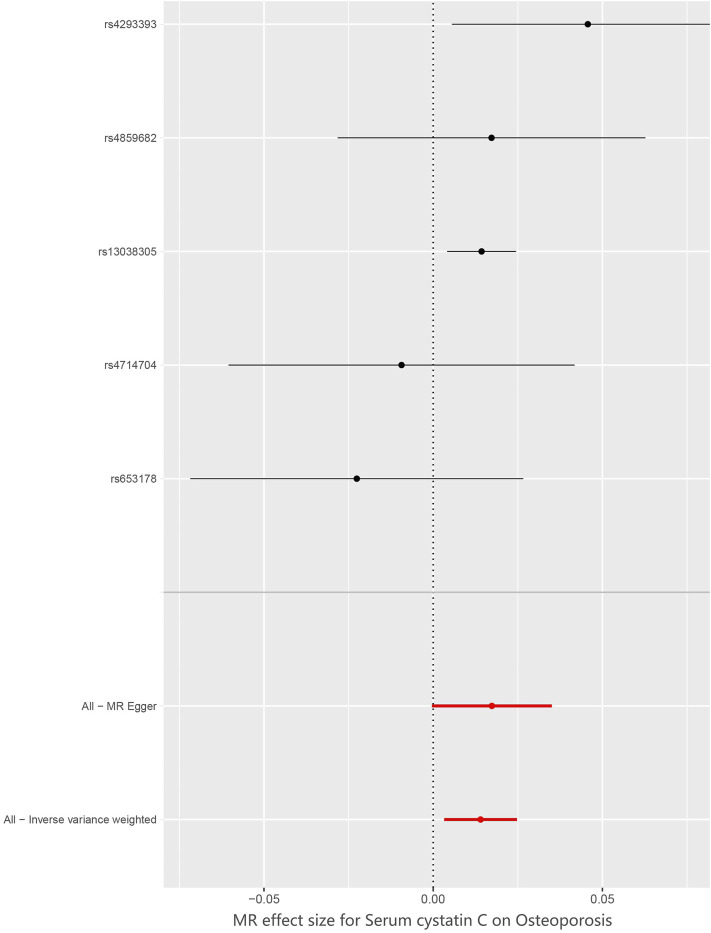
Forest plot to visualize causal effect of each single SNP on osteoporosis risk.

The SNP characteristics of cystatin C and osteoporosis are shown in Table 1. Ultimately, we chose 5 SNPs as IVs (rs653178, rs4293393, rs13038305, rs4859682 and rs4714704). All genetic instruments associated with cystatin C were at the genome-wide significance level (*p* < 5×10^−8^). The strength of the selected single IVs had an F-statistic value between 32 and 926 and *r* ^2^ < 0.001. Therefore, all SNPs were not weak instrumental variables. The causal effects of each genetic variation on osteoporosis are shown in Figures 2, 3.

**TABLE 1:** List of genetic instruments for cystatin C and log odds ratios of osteoporosis risk by each instrumental SNPs (GWAS significance with *p* < 5 × 10^−8^ and linkage disequilibrium. threshold with *R* ^2^ < 0.001).

No.	SNP	Gene	Chr.	EA	OA	EAF.cystatin C	EAF.OP	Cystatin C*β* (SE)	OP*β* (SE)
1	rs13038305	CST3	20	T	C	0.262	0.22077	0.07 (0.0023)	0.001 (0.0004)
2	rs4293393	UMOD	16	G	A	0.177	0.18323	0.019 (0.0023)	0.0009 (0.0004)
3	rs4714704	POLR1C	6	A	G	0.241	0.269003	0.013 (0.0023)	−0.0001 (0.0003)
4	rs4859682	SHROOM3	4	A	C	0.442	0.457608	−0.013 (0.0018)	−0.0002 (0.0003)
5	rs653178	ATXN2	12	T	C	0.562	0.517094	0.012 (0.0019)	−0.0003 (0.0003)

Chr. indicates chromosome; EA, effect allele; OA, other allele; EAF, effect allele frequency; OP, Osteoporosis; SE, standard error.

### 3.2 MR Analyses Results

**FIGURE 4:**
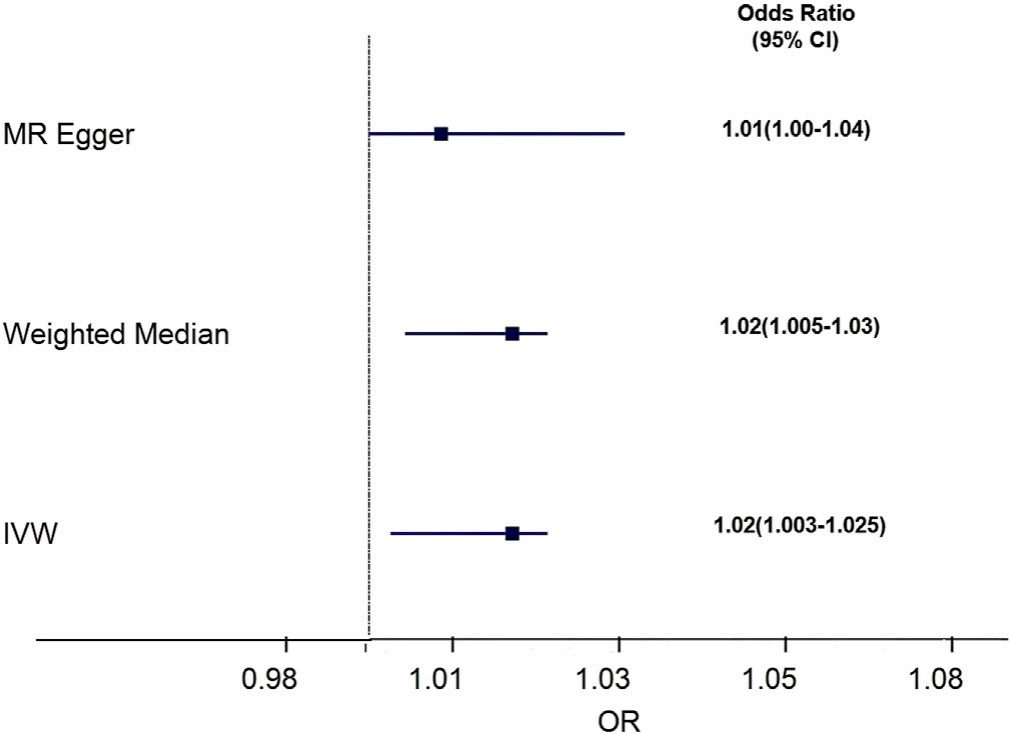
Forest plot to visualize causal effect of Cystatin C on osteoporosis risk. IVW indicates inverse-variance weighted; and MR, Mendelian randomization.

We assessed the causal association between cystatin C levels and osteoporosis patients by using IVW, MR–Egger, and weighted median regressions (Figure 4). Using the IVW method, we found that genetically predicted cystatin C was causally and positively associated with the risk of osteoporosis [OR = 1.02, 95% CI = 1.003–1.025, *p* = 0.01]. Weighted median regression also showed directionally similar estimates [OR = 1.02, 95% CI = 1.005–1.03, *p* = 0.005]. The MR–Egger method showed no significant statistical significance [OR = 1.02, 95% CI = 1.000–1.036, *p* = 0.15]. Fortunately, the estimation directions of the three methods were consistent. For each SNP, no potential pleiotropy was found by using the MR-PRESSO global test (*p* = 0.558).

### 3.3 Sensitivity Analysis

**FIGURE 5:**
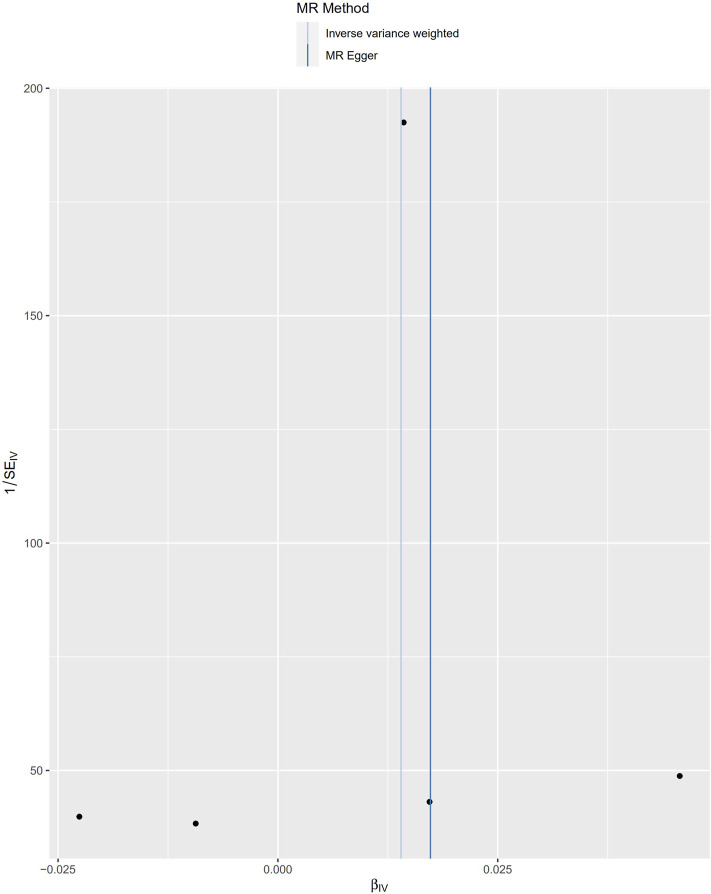
Funnel plots to visualize overall heterogeneity of MR estimates for the effect of Cystatin C on total osteoporosis. IVW indicates inverse-variance weighted; and MR, Mendelian randomization.

Funnel plots can show the directional horizontal pleiotropy of IVs by drawing a single Wald ratio for each SNP. However, due to the small number of IVs included, it is difficult for us to use funnel plots to detect horizontal pleiotropy. The causal effect of the funnel plot was roughly symmetrical (Figure 5), and the intercept of the MR–Egger regression did not observe horizontal pleiotropy (*p* = 0.178), further showing that pleiotropy did not bias the causal effect.

**FIGURE 6:**
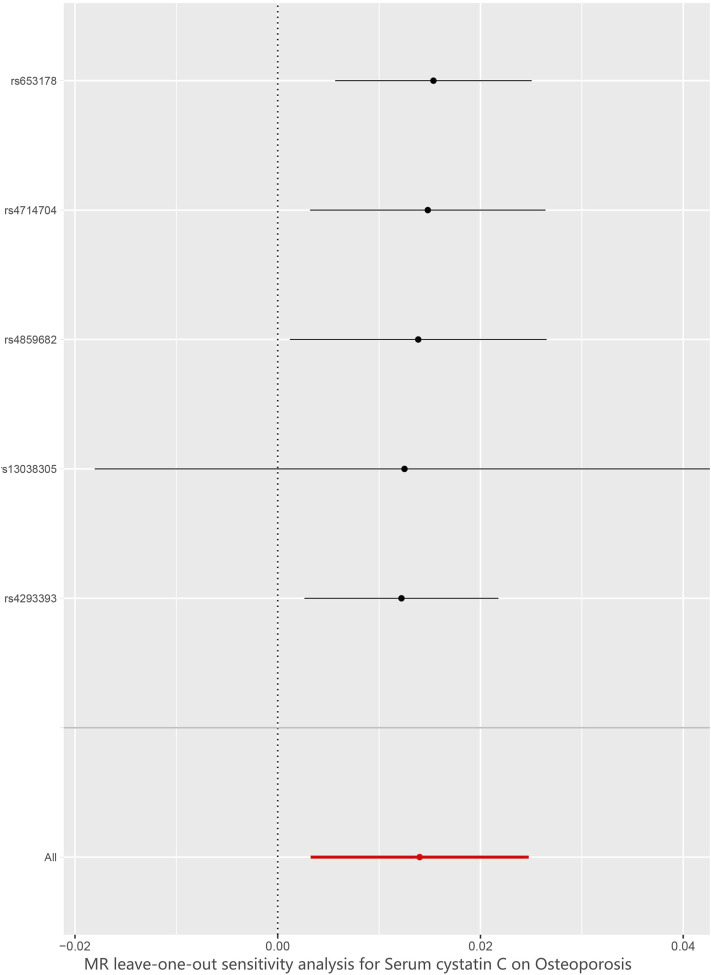
Leave-one-out plot to visualize causal effect of Cystatin C on total osteoporosis risk when leaving one SNP out. MR indicates Mendelian randomization.

We verified the impact of each SNP on the overall causal estimate by leave-one-out analysis. As shown in Figure 6, we systematically performed the MR analysis again on the remaining SNPs after removing each SNP. The results remained consistent, suggesting that the calculation results of all SNPs made causality significant. This also indicates that there was no dominant SNP in cystatin C levels and osteoporosis, and the previous MR results were valid.

## 4 Discussion

This is the first study to explore the causal association between cystatin C levels and osteoporosis risk by a two-sample MR analysis based on a large amount of GWAS data of cystatin C (exposure) and osteoporosis (outcome). This MR study showed that serum cystatin C levels might be causally associated with an increased risk of osteoporosis, and the OR per 0.8-mg/L increase (approximately 1 SD) in serum cystatin C levels was 1.02 for osteoporosis (Stevens et al., 2008).

Cystatin C, a cysteine protease inhibitor, is a protein with 122 amino acids and a relative molecular mass of 13,000. It is a member of a large family of proteins called cysteine protease inhibitors. Cystatin C is different from the conventional diagnostic indicators of renal function injury (urea nitrogen, creatinine, urinary microalbumin, etc.) (Grubb et al., 2014). Cystatin C is found in various body fluids, with the highest concentration in cerebrospinal fluid and the lowest in urine. It can be freely filtered through the glomerulus, is reabsorbed and completely catabolized in the proximal convoluted tubules, and does not return to the blood. Therefore, its concentration in the blood is mainly determined by glomerular filtration. Cystatin C was not affected by age, sex, inflammation, diet, body weight, or liver function (Pucci et al., 2007). Thus, it is an ideal endogenous marker reflecting the glomerular filtration rate and an independent predictor of cardiovascular disease, metabolic syndrome, and diabetes (Hart et al., 2017). Given its biological importance, serum cystatin C is also related to many other diseases.

Osteoporosis is a systemic metabolic bone disease, and its incidence increases with age. It affects the health of approximately 200 million people worldwide (Cooper, 1999). Many observational studies have explored the relationship between cystatin C and osteoporosis. In a prospective case–control study of the Swedish population, Malmgren et al., 2020 found that serum cystatin C was significantly higher in patients with osteoporosis than in normal people, especially in those aged ≥65 years (*p* = 0.043). Kuroda et al. (Kuroda et al., 2013) investigated a population in rural Japan and found that serum cystatin C was significantly associated with osteoporosis and believed that it played an important role in osteoporotic vertebral compression fractures. In a cross-sectional study of Korean adults conducted by Dongwon et al. (Yi et al., 2017), female cystatin C levels were negatively correlated with the bone mineral density of the lumbar spine and femur but were associated with an increased prevalence of osteoporosis. The advantage of serum cystatin C measurement is that bone mineral density can be determined early, which may enable us to prevent osteoporosis and related diseases. However, different research populations may come to different conclusions. Recent prospective studies conducted by Nedeljikovic et al. (Nedeljkovic et al., 2019) in elderly men with chronic heart failure have obtained different results. They believed that there is a positive correlation between cystatin C and bone mineral density in healthy controls and that higher cystatin C was associated with increased bone turnover in elderly men with chronic heart failure by measuring bone metabolic derivatives, suggesting that cystatin C affects the signalling cascade of bone morphogenetic proteins in osteoblasts, thereby promoting bone formation. This is contrary to previous studies, suggesting that cystatin C may play a complicated role in the crosstalk between bones and kidneys (Nedeljkovic et al., 2019).

The results of observational studies further indicate that elevated serum cystatin C levels may be causally related to osteoporosis risk. However, compared with MR analysis, the results of traditional observational studies are more susceptible to reverse causality or other potential confounding effects, and MR analysis may provide the best results for determining causal association (Liu et al., 2021). To date, we have not found any causal association between cystatin C and osteoporosis in MR studies other than ours. We also found similar results to previous observational studies by aggregating statistics on cystatin C (*n* = 33,152) and osteoporosis (*n* = 337,159) from a large sample of GWAS studies. Our study provides direct evidence that genetically determined cystatin C has a causal association with a 2% increase in osteoporosis disease risk, which has been confirmed by IVW and weighted median regression. Although the OR is small, 33% of females and 20% of males over the age of 50 suffer from osteoporosis worldwide (International Osteoporosis Foundation, 2022). Since the osteoporosis population is relatively large, it is necessary to pay attention to the causal relationship between cystatin C and osteoporosis.

To date, the mechanism of action of cystatin C on osteoporosis is still inconclusive. Several mechanisms have been proposed to link cystatin C to the pathogenesis of osteoporosis. Cystatin C is a cysteine protease inhibitor that participates in the body’s metabolism of homocysteine (Hcy). Cystatin C may affect BMD by regulating the metabolism of Hcy. When the level of cystatin C is increased, the decomposition of Hcy is inhibited, and the serum level is increased. Hcy altered vascular dilation and increased vascular resistance by enhancing the oxidative stress response and decreasing the bioavailability of nitric oxide (NO). Ultimately, it reduces blood flow to the bone and promotes bone loss (Kumar et al., 2017). Studies have shown that the activity of osteoblasts decreases with increasing Hcy concentration, and Hcy can activate the activity of osteoclasts and inhibit the apoptosis of osteoclasts. The homeostatic environment is destroyed by affecting the balance between bone resorption and bone formation (Behera et al., 2018). Tanaka et al. (Tanaka et al., 2019) believed that cystatin C directly acts on osteoclast precursor cells through the intracellular mechanism of the RANK pathway. In osteoporosis, osteoclast differentiation is enhanced, and cystatin C is not taken up by osteoclast precursor cells, which leads to an increase in serum cystatin C levels. In addition, osteoclasts specifically express cysteine proteases, and the important part of their binding site is the amino terminus of cystatin C (Brage et al., 2004). Their combination reduced osteoclast formation and the expression of RANK. Hence, the secondary elevation of cystatin C effectively inhibited bone resorption. It has also been reported that even in mild kidney injury, serum cystatin levels increase, 1,25-dihydroxy vitamin D synthesis decreases, calcium absorption decreases, and secondary hyperparathyroidism eventually leads to bone loss (Vieth et al., 2003). Other studies suggest that cystatin C can also cause an inflammatory response and increase C-reactive protein indirectly through a chronic inflammatory response to participate in the formation of osteoporosis (Keller et al., 2008). But, the specific mechanism of cystatin C needs to be further clarified.

Our study has several major strengths. To the best of our knowledge, this is the first MR to explore the causal association between cystatin C and osteoporosis. MR is similar in concept to prospective randomized controlled trials (RCTs) but reduces systematic biases that affect the results of traditional observational studies, such as confounding factors and reverse causality. The high accuracy of genotyping can effectively avoid regression dilution caused by detection errors. To ensure that SNPs are not related to any confounding factors between cystatin C and osteoporosis, we chose only participants from European populations. Finally, to ensure the stability of the results, we also performed MR-PRESSO and MR–Egger regression tests, and no evidence of directional level pleiotropy was observed.

There are limitations in this MR. First, we used aggregated GWAS data, and the lack of specific information on sex and age prevented us from conducting subgroup analysis. Second, we only included European GWASs and cannot determine whether the results of this study also apply to Asians because genetic variations exist between different races (Tan et al., 2021b). Third, although we obtained a small OR, focusing on the causal relationship between cystatin C and osteoporosis is warranted because of the large prevalence. Fourth, there may be a false-positive rate between different methods, which is difficult to calculate. Finally, the power we calculated by using mRnd (https://shiny.cnsgenomics.com/mRnd/) was only 10%, suggesting that our MR study had low statistical power, mainly due to the insufficient sample size of the included studies. This finding needs to be further verified in a study with a larger sample size.

## 5 Conclusion

In conclusion, our MR study showed evidence of a causal association between serum cystatin C levels and osteoporosis, and the results need to be verified in studies with larger sample sizes in the future. Early monitoring of cystatin C may enable us to prevent osteoporosis-related diseases.

## Data Availability

The original contributions presented in the study are included in the article/Supplementary Material, further inquiries can be directed to the corresponding authors.
